# Associations between negative life events and depressive symptoms in Chinese adolescents: the mediating role of self-esteem and coping tendency

**DOI:** 10.3389/fpsyg.2026.1826479

**Published:** 2026-06-25

**Authors:** Yifan Zhang, Juntao Chen, Shiqi Huang, Peizheng Li, Shihong Song, Xiyuan Fu, Jiaxin Liao, Weiping Wang, Lu Ma, Xiaoming Huang

**Affiliations:** 1 Beijing Anzhen Nanchong Hospital of Capital Medical University & Nanchong Central Hospital, Nanchong, Sichuan, China; 2 Department of Epidemiology and Biostatistics, School of Public Health, Wuhan University, Wuhan, Hubei, China; 3 Department of Safety and Environmental Protection, China Nuclear Power Engineering Co., Ltd., Shenzhen, Guangdong, China

**Keywords:** adolescents, chain mediation, coping tendency, depression symptoms, negative life events, self-esteem

## Abstract

**Background:**

Numerous studies have highlighted the links between depressive symptoms, negative life events, self-esteem, and coping tendency in adolescents. However, the intricate associations and potential pathways to depressive symptoms remain unclear. This study aimed to explore these pathways using a theoretically driven chain-mediated approach.

**Methods:**

A cross-sectional survey was conducted among 2,805 junior high school students in two cities in Hubei Province, China. Participants completed scales assessing negative life events, self-esteem, coping tendency, and depressive symptoms. Structural equation modeling (SEM) adjusted for the complex survey design was employed to test the sequential indirect effects of self-esteem and coping tendency between negative life events and depressive symptoms. Multi-group SEM was conducted to examine whether indirect effects differed by gender and grade.

**Results:**

Greater exposure to negative life events was associated with more severe depressive symptoms ( β = 0.57, 95% CI: 0.48, 0.65), lower self-esteem ( β = − 0.49, 95% CI: −0.55, −0.44), and a less positive coping tendency ( β = − 0.16, 95% CI: −0.20, −0.12). Higher self-esteem was associated with a more positive coping tendency ( β = 0.49, 95% CI: 0.44, 0.55) and less severe depressive symptoms ( β = − 0.22, 95% CI: −0.29, −0.15). A more positive coping tendency was associated with less severe depressive symptoms ( β = − 0.11, 95% CI: −0.15, −0.07). The estimated total indirect association through self-esteem and coping tendency was 0.15 (95% CI: 0.12, 0.19), accounting for 21.2% of the total association, and comprised three components: the path through self-esteem alone (0.11, 15.0%), through coping tendency alone (0.02, 2.5%), and sequentially through both (0.03, 3.6%). These indirect associations did not differ significantly by gender or grade.

**Conclusion:**

Higher self-esteem and a more positive coping tendency showed a sequential indirect association linking negative life events to depressive symptoms, suggesting they may be potential intervention targets. Cross-sectional data preclude causal inference; longitudinal studies are needed to verify the proposed pathway.

## 1 Introduction

Depression has posed a serious challenge to the adolescent population, as it not only affects an individual’s emotional and cognitive development (Racine et al., 2021; Malhi and Mann, 2018), but also leads to a range of problems, including reduced quality of life (Berlim and Fleck, 2007), reduced academic productivity (Afzali et al., 2018), and strained relationships (Santini et al., 2015), among others. In China, the 2022 Report on National Adolescent Mental Health reported that 14.8% of adolescents were at risk of depression (Fu et al., 2023). Separately, a meta-analysis of 51 studies estimated that approximately 24.3% of secondary school students experience depressive symptoms (Tang et al., 2019). Trend analyses spanning 1989 to 2018 further indicate that the prevalence of depressive symptoms among Chinese adolescents has increased over recent decades (Su and Liu, 2022). Adolescent depression has thus become a pressing public health concern.

The causes of depression and the emergence of associated symptoms are complex. Adolescents are at a developmental stage during which their moods and emotions are particularly vulnerable to external stressors and internal psychological factors (Platt et al., 2021). Negative life events act as external disturbances in adolescents’ lives, including interpersonal conflicts, academic stress, health problems and adjustment problems (Liu et al., 1997). Frequent encounters with such challenges can directly induce depressive symptoms in adolescents (Geng et al., 2022; Sarbu et al., 2022; Boe et al., 2018; Stikkelbroek et al., 2016), manifesting physiological dysfunction (He et al., 2023; Horesh et al., 1995), and deterioration of psychological state (Kraaij et al., 2003). Self-esteem and coping tendency, two factors that reflect an individual’s psychological attributes (Baumeister et al., 2003; Folkman and Moskowitz, 2004), play a complex and critical role in the development of depression. Research has shown that low self-esteem is often associated with an increased risk of depression (Sowislo and Orth, 2013; Yeo et al., 2023), while a tendency toward negative coping is associated with poor mental health outcomes (Compas et al., 2017; Lewis et al., 2012; Nagase et al., 2009). In addition, negative life events have also been associated with lower self-esteem and the adoption of negative coping strategies (Baldwin and Hoffmann, 2002; Tetzner et al., 2016; Veisani et al., 2021), suggesting that self-esteem and coping tendency may serve as intermediate psychological processes linking negative life events to depressive symptoms.

Theoretically, the proposed sequential ordering of self-esteem and coping tendency can be grounded in Lazarus’s cognitive appraisal theory of stress and coping (Folkman, 2013). According to this framework, when individuals encounter stressors, they first engage in primary appraisal, during which core self-schemas including self-esteem are activated to evaluate the personal significance of the event. Self-esteem functions as a cognitive filter that shapes how stressors are interpreted, such that adolescents with lower self-esteem are more likely to appraise negative events as reflections of personal inadequacy. This primary appraisal, in turn, triggers secondary appraisal, which involves the evaluation of available coping resources and options and subsequently guides the selection of coping strategies. Coping strategies represent more proximal, situation-specific behavioral responses that directly regulate emotional outcomes, whereas self-esteem reflects a relatively stable trait that influences depression indirectly through its effect on coping. Thus, self-esteem is positioned as the more distal mediator and coping tendency as the more proximal one in the pathway to depressive symptoms. This proposed ordering is derived from theoretical considerations rather than from temporal precedence in the data, as all variables were measured concurrently in the present cross-sectional design.

Based on this theoretical framework and the empirical evidence reviewed above, the present study aimed to examine the hypothesized sequential indirect pathway linking negative life events, self-esteem, coping tendency, and depressive symptoms among Chinese junior high school students. Specifically, we tested four hypotheses: (1) a direct association between negative life events and depressive symptoms; (2) an indirect association via self-esteem, such that greater exposure to negative life events is associated with lower self-esteem, which in turn is associated with more severe depressive symptoms; (3) an indirect association via coping tendency, such that negative life events are associated with a less positive coping tendency, which in turn is negatively associated with depressive symptoms; and (4) a sequential indirect association, such that negative life events are associated with lower self-esteem, which in turn is associated with a less positive coping tendency, which in turn is associated with higher depressive symptoms. In addition, we explored whether these indirect associations differed by gender and grade.

## 2 Materials and methods

### 2.1 Design and procedure

This study was conducted as part of an intervention programme on the mental health of left-behind children in Tongcheng County and Lichuan City, both of which are relatively underdeveloped areas in Hubei Province, China. Aligned with the program’s focus on left-behind children, the sample size was determined based on this population, with their regional proportion serving as a stratification criterion. The study used a multi-stage proportional probability sampling (PPS) design. Schools were stratified according to the type of school (public or private) and the administrative region. Within these strata, schools were sampled with probabilities proportional to their size. Subsequently, the proportion of left-behind children in each selected school was used as a secondary stratification criterion to adjust the required quota of classes. Finally, classes were randomly selected from the targeted grades in the sampled schools, and all students within these classes were invited to participate.

After receiving uniform training, all participating staff travelled to the schools to distribute the electronic or paper questionnaires, with two to three enumerators per school. Respondents were instructed to complete the questionnaires independently to ensure authenticity and reliability. The study was reviewed and approved by the Ethics Committee of Wuhan University (Approval No. WHU-HSS-IRB2023019). Written informed consent was obtained from parents or legal guardians, and assent was obtained from the students themselves prior to participation.

Data collection occurred between May 17 and May 25, 2023, yielding 2,909 questionnaires. Questionnaires with any missing values across the core scales, demographic covariates, or survey design variables were excluded, retaining 2,805 valid responses (96.4%). Excluded and retained respondents did not differ on primary study variables (all *p* > 0.05). Full attrition results are provided in Supplementary Table S1.

### 2.2 Measurement

#### 2.2.1 Socio-demographic characteristics

A self-compiled general information questionnaire is employed to gather data on socio-demographic characteristics, including age, gender (boys/girls), grade level (7th/8th grade), ethnicity (ethnic han/minority), left-behind status (yes/no), only-child status (yes/no), boarding status (yes/no), parents’ marital status (married/other), and perceived family income (upper/middle/lower).

#### 2.2.2 Negative life events

The Adolescent Self-Assessed Negative Life Events Checklist (ASLEC) was used to assess whether or not a negative event has been experienced in an adolescent’s life in the past year and the extent to which this event has had an impact on psychological wellbeing (Liu et al., 1997). The ASLEC scale consists of 27 negative life events that may be psychologically burdensome for adolescents and covers areas such as family, school, and social relationships. For each event, participants indicated whether it occurred; if it did, they rated its impact on a 5-point scale (1 = not at all, 2 = mildly, 3 = moderately, 4 = severely, 5 = extremely). Events that did not occur were scored 0. Total scores range from 0 to 135, with higher scores reflecting greater cumulative stress. The Cronbach’s *α* for the ASLEC in the present study was 0.94.

#### 2.2.3 Self-esteem

Self-esteem was measured using the Rosenberg Self-Esteem Scale (RSES), which assesses adolescents’ global feelings of self-worth and self-acceptance (Rosenberg, 1965). The instrument consists of 10 items (e.g., “I feel I am a capable person”), five positively worded and five negatively worded, each rated on a 4-point scale (1 = strongly disagree, 2 = disagree, 3 = agree, 4 = strongly agree). Negatively worded items were reverse-scored, yielding total scores from 10 to 40, with higher scores indicating greater self-esteem. The Cronbach’s *α* for the RSES in the present study was 0.88.

#### 2.2.4 Coping tendency

Coping tendency was measured using the Simplified Coping Style Questionnaire (SCSQ), a Chinese adaptation of Lazarus’s coping framework (Folkman and Lazarus, 1988; Xie, 1998; Fang et al., 2018). The SCSQ comprises 20 items divided into two dimensions: positive coping (items 1–12) and negative coping (items 13–20). Each item is rated on a 4-point scale (0 = never, 1 = occasionally, 2 = sometimes, 3 = often). A coping tendency score was calculated as the difference between the standardized positive coping score and the standardized negative coping score. A positive score indicates a tendency toward positive coping, a negative score indicates a tendency toward negative coping, and higher scores consistently reflect a greater relative preference for positive over negative coping strategies. The Cronbach’s *α* in this study was 0.89 for the positive coping subscale and 0.77 for the negative coping subscale.

#### 2.2.5 Depression symptoms

Depressive symptoms were assessed using the Patient Health Questionnaire-9 (PHQ-9) (Kroenke et al., 2001), which was designed to assess the presence and severity of depressive symptoms in individuals based on the DSM-IV criteria for the diagnosis of depression. The PHQ-9 consists of 9 items covering depressed mood, anhedonia, sleep disturbances, fatigue, appetite changes, feelings of worthlessness, concentration difficulties, psychomotor changes, and suicidal ideation. Participants rated the frequency of each symptom over the past 2 weeks on a 4-point scale (0 = not at all, 1 = several days, 2 = more than half the days, 3 = nearly every day). Total scores range from 0 to 27, with higher scores indicating greater symptom severity. Based on the established severity classification, a total score ≥ 10 was used to identify the presence of moderate-to-severe depressive symptoms (Kroenke et al., 2001). The Chinese version of the PHQ-9 has demonstrated adequate reliability and validity in Chinese adolescent samples (Leung et al., 2020). The Cronbach’s *α* of the PHQ-9 in this study was 0.91.

### 2.3 Statistical analyses

To account for the multi-stage PPS design—in which students were nested within classes and schools—a complex survey design object was constructed with schools and classes as clustering units, stratified by administrative region, and weighted by inverse selection probabilities. Standard errors and model parameters were adjusted via Taylor series linearization using the lavaan.survey package. Scores on the four core scales (depressive symptoms, negative life events, self-esteem, and coping tendency) were summarized as medians with interquartile ranges (P_25_, P_75_) and compared across demographic subgroups using design-adjusted Mann–Whitney U or Kruskal-Wallis tests. Bivariate associations among the four core variables were assessed with design-weighted Spearman correlations.

The hypothesized chain mediation model was tested using structural equation modeling (SEM) in lavaan. All continuous variables were standardized prior to analysis. The model specified self-esteem and coping tendency as sequential mediators linking negative life events to depressive symptoms, with age, gender, grade, ethnicity, boarding status, left-behind status, only-child status, parents’ marital status and family income included as covariates in all three regression equations. Three specific indirect effects were defined: (1) negative life events → self-esteem → depressive symptoms (Ind1); (2) negative life events → coping tendency → depressive symptoms (Ind2); and (3) negative life events → self-esteem → coping tendency → depressive symptoms (Ind3). The total indirect effect was calculated as Ind1 + Ind2 + Ind3. Statistical significance of indirect effects was assessed via survey-adjusted 95% confidence intervals; an effect was considered significant if its interval excluded zero.

To test whether the indirect effects differed by gender and grade, multi-group SEM was conducted with group-specific path coefficients. The grouping variable was excluded from the covariate set in each respective model, while all other covariates were retained as fixed effects. Group differences in each indirect effect were directly tested via contrast parameters, with significance determined by whether the survey-adjusted 95% confidence interval (95% CI) for the contrast parameter excluded zero.

Common method bias was assessed within the complex survey framework using parcel-based confirmatory factor analysis (CFA). Items for each of the four core constructs were randomly allocated into three parcels, yielding 12 indicators. A one-factor model (all 12 parcels loading onto a single common factor) was compared against the hypothesized four-factor model using the robust maximum likelihood estimator. Model fit was evaluated using the scaled chi-square statistic, robust comparative fit index (CFI), robust Tucker-Lewis index (TLI), and robust root mean square error of approximation (RMSEA). The two models were compared using the Satorra-Bentler scaled chi-square difference test.

All statistical analyses were performed in R version 4.4.3. All tests were two-tailed, and statistical significance was set at *α* = 0.05.

## 3 Results

### 3.1 Common method biases tests

A single-factor CFA model (all 12 item parcels loading onto one common factor) was compared against the hypothesized four-factor model. The single-factor model exhibited poor fit (CFI = 0.634, TLI = 0.553, RMSEA = 0.251), whereas the four-factor model fitted adequately (CFI = 0.981, TLI = 0.974, RMSEA = 0.060). The four-factor model provided a significantly better fit (Satorra-Bentler $\Delta \chi^{2}$ (6) = 8533.8, *p* < 0.05), indicating that common method bias does not predominantly drive the observed associations.

### 3.2 Descriptive statistics and demographic comparisons

Among the 2,805 participants, the weighted prevalence of moderate-to-severe depressive symptoms (PHQ-9 ≥ 10) was 21.6% (95% CI: 18.8, 24.8%). The sample comprised 1,474 boys (52.4%) and 1,331 girls (47.6%). The mean age was 13.5 ± 0.8 years, with most participants aged 13–14 years. Table 1 presents the design-adjusted descriptive statistics for all core variables across demographic subgroups. Depressive symptom scores differed significantly by gender, grade, left-behind status, parents’ marital status, and perceived family income (all *p* < 0.05), but did not differ by ethnicity, only-child status, or boarding status. Negative life events, self-esteem, and coping tendency scores also showed significant variations across most demographic subgroups.

**Table 1:** Descriptive statistics and demographic comparisons of study variables across subgroups (*N* = 2,805).

Variables	*N* (%)	Depression symptoms		Negative life events		Self-esteem		Coping tendency	
		Md (P_25_, P_75_)	*p*	Md (P_25_, P_75_)	*p*	Md (P_25_, P_75_)	*p*	Md (P_25_, P_75_)	*p*
Full sample	2,805 (100.0)	5 (1, 9)		17 (8, 32)		27 (24, 31)		0.00 (−0.89,0.95)	
Gender			<0.001		<0.001		<0.001		0.333
Boys	1,474 (52.4)	4 (1, 8)		16 (8, 31)		28 (25, 31)		0.02 (−0.86, 0.98)	
Girls	1,331 (47.6)	5 (2, 10)		19 (10, 34)		27 (23, 30)		−0.03 (−0.90, 0.90)	
Grade			<0.001		<0.001		0.003		<0.001
7th	1,344 (49.8)	3 (1, 7)		14 (7, 26)		28 (25, 32)		0.34 (−0.61, 1.29)	
8th	1,461 (50.2)	6 (2, 10)		22 (11, 39)		26 (23, 30)		−0.35 (−1.07, 0.60)	
Ethnicity			0.542		0.759		0.123		0.022
Ethnic Han	1,340 (45.3)	4 (1, 9)		17 (8, 31)		27 (24, 30)		−0.18 (−0.93, 0.78)	
Minority	1,465 (54.7)	5 (1, 9)		17 (8, 32)		27 (24, 31)		0.12 (−0.80, 1.10)	
Left−behind			0.006		0.009		0.004		0.006
No	1,293 (47.2)	4 (1, 8)		15 (8, 29)		28 (24, 32)		0.12 (−0.80, 1.11)	
Yes	1,512 (52.8)	5 (2, 9)		19 (10, 34)		27 (23, 30)		−0.05 (−0.93, 0.82)	
Only child			0.542		0.188		0.317		0.583
No	2,385 (84.9)	5 (1, 9)		18 (8, 33)		27 (24, 31)		0.02 (−0.90, 0.95)	
Yes	420 (15.1)	4 (1, 9)		16 (8, 30)		27 (24, 31)		−0.13 (−0.84, 0.91)	
Boarding			0.971		0.826		0.407		0.420
No	895 (31.1)	5 (1, 9)		17 (8, 33)		27 (23, 31)		−0.08 (−0.93, 0.81)	
Yes	1910 (68.9)	4 (1, 9)		17 (8, 32)		27 (24, 31)		0.03 (−0.84, 0.98)	
Parents’ marital status			0.004		<0.001		0.025		0.007
Married	2,289 (81.6)	4 (1, 8)		16 (8, 31)		27 (24, 31)		0.06 (−0.82, 0.98)	
Other	516 (18.4)	6 (2, 11)		20 (11, 37)		26 (23, 30)		−0.34 (−1.05, 0.73)	
Perceived family income			<0.001		<0.001		<0.001		<0.001
Upper	390 (15.0)	4 (1, 8)		14 (7, 31)		29 (25, 33)		0.14 (−0.74, 1.28)	
Middle	2055 (73.5)	4 (1, 8)		16 (8, 30)		27 (24, 31)		0.04 (−0.81, 0.94)	
Lower	360 (11.5)	8 (3, 13)		27 (14, 47)		25 (21, 28)		−0.57 (−1.27, 0.40)	

Unweighted N and design-weighted percentages are presented. *p*-values were derived from design-based Wilcoxon rank-sum or Kruskal-Wallis tests.

### 3.3 Correlation analyses

Design-weighted Spearman correlations among the four core variables were all statistically significant (all *p* < 0.05). Depressive symptoms were positively correlated with negative life events (*r* = 0.75) and negatively correlated with self-esteem (*r* = −0.61) and coping tendency (*r* = −0.49). Negative life events were negatively correlated with self-esteem (*r* = −0.54) and coping tendency (*r* = −0.43). Self-esteem was positively correlated with coping tendency (*r* = 0.58).

### 3.4 The chain mediation effects analyses

**Figure 1:**
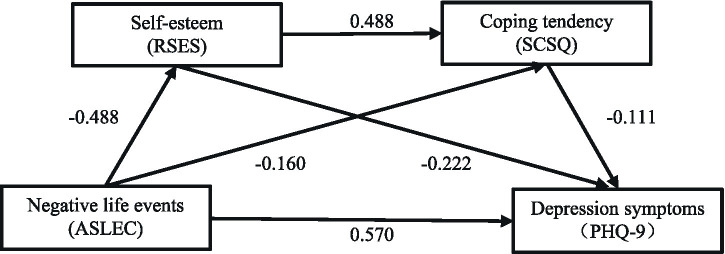
The hypothesized chain mediation model linking negative life events, self-esteem, coping tendency, and depressive symptoms. Path coefficients are standardized estimates from the survey-adjusted SEM. All displayed paths were statistically significant.

The chain mediation model was tested with negative life events as the independent variable, self-esteem and coping tendency as sequential mediators, and depressive symptoms as the dependent variable, adjusting for all demographic covariates. All three specific indirect effects were statistically significant (Table 2). The indirect effect through self-esteem alone was 0.11 (95% CI: 0.08, 0.14). The indirect effect through coping tendency alone was 0.02 (95% CI: 0.01, 0.03). The chain mediation effect through both mediators was 0.03 (95% CI: 0.02, 0.04). The total indirect effect was 0.15 (95% CI: 0.12, 0.19), accounting for 21.2% of the total effect (0.72; 95% CI: 0.65, 0.78). Full path coefficients for all three regression equations are presented in Table 3. The hypothesized chain mediation model is depicted in Figure 1.

**Table 2:** Indirect effects from the survey-adjusted chain mediation model.

Indirect path	Estimate	SE	95% CI	Proportion mediated
Ind1: NLE → SE → DEP	0.108	0.014	0.079, 0.136	15.04%
Ind2: NLE → COP → DEP	0.018	0.004	0.010, 0.025	2.51%
Ind3: NLE → SE → COP → DEP	0.026	0.006	0.015, 0.037	3.62%
Total indirect	0.152	0.018	0.116, 0.187	21.17%
Direct effect (*c*’)	0.567	0.043	0.482, 0.652	−
Total effect	0.718	0.034	0.652, 0.784	−

NLE, negative life events; SE, self-esteem; COP, coping tendency; DEP, depressive symptoms. Estimate, point estimate of the indirect effect; SE, design-adjusted standard error; 95% CI, survey-adjusted Wald confidence interval. Proportion mediated = indirect effect/total effect × 100%. All coefficients are adjusted for age, gender, grade, ethnicity, boarding status, left-behind status, only-child status, parents’ marital status, and family income.

**Table 3:** Path coefficients from the survey-adjusted chain mediation model.

Path	B	SE	β	*Z*	*p*	95% CI
Negative life events → Self-esteem (*a*_1_)	−0.492	0.027	−0.488	−18.22	< 0.001	−0.545, −0.438
Negative life events → Coping tendency (*a*_2_)	−0.162	0.020	−0.160	−8.10	< 0.001	−0.201, −0.123
Self-esteem → Coping tendency (*d*_21_)	0.491	0.029	0.488	16.93	< 0.001	0.435, 0.547
Negative life events → Depression symptoms (*c*’)	0.567	0.043	0.570	13.19	< 0.001	0.482, 0.652
Self-esteem → Depression symptoms (*b*_1_)	−0.219	0.035	−0.222	−6.26	< 0.001	−0.288, −0.150
Coping tendency → Depression symptoms (*b*_2_)	−0.109	0.018	−0.111	−6.06	< 0.001	−0.145, −0.073

B, unstandardized coefficient; SE, design-adjusted standard error; β , standardized coefficient (all variables were standardized prior to analysis); Z = Z-statistic (B/SE); 95% CI, survey-adjusted Wald confidence interval. All coefficients are adjusted for age, gender, grade, ethnicity, boarding status, left-behind status, only-child status, parents’ marital status, and family income.

### 3.5 Multi-group comparisons by gender and grade

Multi-group SEM was conducted to test whether the indirect effects differed by gender and grade (Table 4). All three indirect paths were significant within each subgroup. However, no group differences reached statistical significance. For gender, the total indirect effect was 0.12 (95% CI: 0.09, 0.16) for boys and 0.19 (95% CI: 0.13, 0.24) for girls, with a non-significant difference of −0.06 (95% CI: −0.13, 0.00). For grade, the total indirect effect was 0.18 (95% CI: 0.14, 0.22) for 7th graders and 0.13 (95% CI: 0.08, 0.19) for 8th graders, with a non-significant difference of 0.05 (95% CI: −0.02, 0.11).

**Table 4:** Conditional indirect effects and multi-group comparisons by gender and grade.

Panel A: gender				
Indirect path	Boys (95% CI)	Girls (95% CI)	Δ (Boys − Girls)	95% CI of Δ
Ind1: NLE → SE → DEP	0.089 (0.054, 0.124)	0.128 (0.087, 0.168)	−0.039	−0.092, 0.015
Ind2: NLE → COP → DEP	0.013 (0.006, 0.021)	0.024 (0.008, 0.040)	−0.011	−0.029, 0.007
Ind3: NLE → SE → COP → DEP	0.021 (0.008, 0.033)	0.034 (0.012, 0.056)	−0.014	−0.038, 0.011
Total indirect	0.123 (0.090, 0.156)	0.186 (0.128, 0.244)	−0.063	−0.130, 0.004

Panel B: grade				
Indirect path	7th grade (95% CI)	8th grade (95% CI)	Δ (7th − 8th)	95% CI of Δ
Ind1: NLE → SE → DEP	0.138 (0.114, 0.162)	0.087 (0.043, 0.132)	0.050	−0.001, 0.101
Ind2: NLE → COP → DEP	0.018 (0.008, 0.028)	0.017 (0.004, 0.031)	0.001	−0.016, 0.018
Ind3: NLE → SE → COP → DEP	0.023 (0.010, 0.037)	0.027 (0.012, 0.041)	−0.003	−0.023, 0.016
Total indirect	0.179 (0.142, 0.216)	0.131 (0.076, 0.186)	0.048	−0.018, 0.114

NLE, negative life events; SE, self-esteem; COP, coping tendency; DEP, depressive symptoms. 95% CI, survey-adjusted Wald confidence interval. Δ, group difference. In Panel A, models are adjusted for grade, ethnicity, boarding status, left-behind status, only-child status, parents’ marital status, family income, and age (gender is the grouping variable and is therefore excluded from the covariate set). In Panel B, models are adjusted for gender, ethnicity, boarding status, left-behind status, only-child status, parents’ marital status, family income, and age (grade is the grouping variable and is therefore excluded from the covariate set). All covariates were treated as fixed effects across groups.

## 4 Discussion

The present study found that greater exposure to negative life events, lower self-esteem, and a less positive coping tendency were each associated with more severe depressive symptoms, and that self-esteem and coping tendency showed a sequential indirect association linking negative life events to depressive symptoms. This sequential pattern aligns with the cognitive appraisal framework. According to this theory, external stressors activate core self-schemas, lowering self-esteem, which in turn shapes the selection of coping strategies, ultimately influencing depressive symptoms. However, this theoretical sequence was not empirically demonstrated in the present cross-sectional data. These findings should therefore be interpreted as theoretically informed statistical associations rather than demonstrated causal sequences.

The independent indirect paths through self-esteem and coping tendency are consistent with prior findings (Li et al., 2013; Sawyer et al., 2009). Adolescents with higher self-esteem appear to possess a psychological advantage when facing stressful circumstances; they tend to view themselves more favorably (Brown et al., 2001), a characteristic associated with stronger self-efficacy. Individuals with higher self-efficacy tend to adopt more positive and adaptive coping strategies, whereas those with lower self-efficacy may be more likely to adopt negative or avoidant coping strategies, which in turn affects the trajectory of depressive symptoms (Liu and Yang, 2019; Bandura, 1997). However, research on this topic is not entirely consistent. A study of patients with affective disorders reported that self-esteem fully mediated the link between positive but not negative life events and depressive symptoms, suggesting that the mediating role of self-esteem may vary across populations, particularly between clinical samples and community-based adolescents (Sarubin et al., 2020). In addition, a study of Chinese boarding school students concluded that positive coping moderated rather than mediated the relationship between negative life events and depression (Gao et al., 2021). Wadsworth et al. (2005) argued that coping acts as a mediator in children and adolescents but as a moderator in adults, implying that coping strategies are less crystallized in younger populations and thus function as pathways through which stressors affect mental health. The present findings are compatible with this developmental perspective, suggesting that coping tendency may function as an intermediate process in early adolescence.

Multi-group SEM indicated that none of the indirect effects differed significantly between boys and girls, or between 7th and 8th graders. Prior research has documented that adolescent girls tend to report higher depressive symptoms than boys (Ma et al., 2020; Salk et al., 2016). This pattern has been linked to multiple factors, including gendered socialization processes that may encourage girls to internalize distress, whereas boys may be socialized to externalize or distract from emotional difficulties (Leadbeater et al., 1999; Rose and Rudolph, 2006). Similarly, the transition into secondary school can temporarily heighten psychological vulnerability among 7th graders, who must adapt to increased academic demands and a new social ecology (Shalayiding et al., 2024), whereas 8th graders may have developed more robust coping resources after a year of adaptation (Anyan and Hjemdal, 2016). The absence of significant subgroup moderation in the present study may partly reflect the uniformly high academic pressures and shared campus environment that characterize this sample. In such settings, the dominant environmental stressor may affect students through similar psychological pathways across subgroups, potentially limiting the observable contrast between groups (Tang et al., 2020; Zhu et al., 2021). It is also possible that subgroup differences are more pronounced in clinical or older adolescent populations than in the present community-based sample (Lewis et al., 2020; Musliner et al., 2016).

### 4.1 Limitations

Several limitations should be acknowledged. First, the cross-sectional design precludes causal inference. The chain mediation model assumes a specific temporal ordering based on Lazarus’s cognitive appraisal theory; however, all variables were measured at a single time point, and the measures differed in their retrospective time frames—negative life events were assessed over the past year, depressive symptoms over the past 2 weeks, and self-esteem and coping tendency as current general states without a specified window. This temporal misalignment means that the observed indirect associations may reflect concurrent rather than sequential patterns. Longitudinal studies with repeated measurements and aligned time lags are needed to verify the proposed sequence.

Second, all data were collected via self-report, which may introduce response biases and common method variance. Although parcel-based confirmatory factor analysis suggested that common method bias did not predominantly drive the observed associations, future research should incorporate multi-source assessments, such as parent or teacher reports.

Third, the sample was drawn from two economically underdeveloped counties in Hubei Province, which may limit the generalizability of findings to adolescents in urban or more affluent regions.

## 5 Conclusion

The present study found that greater exposure to negative life events was associated with more severe depressive symptoms among Chinese adolescents, and that self-esteem and coping tendency showed a sequential indirect association linking negative life events to depressive symptoms. These findings suggest that self-esteem and coping tendency may represent potential intervention targets for school-based mental health programs aimed at mitigating the psychological impact of negative life events. However, the cross-sectional design precludes causal inference, and the proposed sequential ordering of mediators requires verification in longitudinal studies with repeated measures and aligned time lags. Future research should also incorporate more diverse samples to strengthen the generalizability of these findings.

## Data Availability

The datasets presented in this article are not readily available because the raw data supporting the conclusions of this article contain sensitive information from a minor population and cannot be shared publicly due to ethical restrictions. De-identified data may be made available upon reasonable request to the corresponding author, subject to ethical approval and institutional data access requirements. Requests to access the datasets should be directed to LM, malu@whu.edu.cn.
