# Supplementary material for: Associations between negative life events and depressive symptoms in Chinese adolescents: the mediating role of self-esteem and coping tendency
Source: Front Psychol. 2026 Jun 25;17:1826479. doi: 10.3389/fpsyg.2026.1826479 (PMC13346051; doi:10.3389/fpsyg.2026.1826479)
Supplement: Supplementary file 1 [file Table_1.docx]

***Supplementary Material***

**Supplementary Tabel 1.** Comparison of socio-demographic characteristics and core study variables between excluded and included participants.

|  | | Excluded  (*N* = 104) | Included  (*N* = 2805) | *P* |
| --- | --- | --- | --- | --- |
| Age (mean [SD]) | | 13.45 [0.85] | 13.49 [0.77] | 0.640 |
| Gender (%) | |  |  | 0.378 |
|  | Boys | 57 (57.6) | 1474 (52.5) |  |
|  | Girls | 42 (42.4) | 1331 (47.5) |  |
| Grade (%) | |  |  | 0.609 |
|  | 7th | 53 (51.0) | 1344 (47.9) |  |
|  | 8th | 51 (49.0) | 1461 (52.1) |  |
| Ethnicity (%) | |  |  | 0.041 |
|  | Ethnic Han | 56 (58.9) | 1340 (47.8) |  |
|  | Minority | 39 (41.1) | 1465 (52.2) |  |
| Left-behind (%) | |  |  | 1.000 |
|  | No | 48 (46.2) | 1293 (46.1) |  |
|  | Yes | 56 (53.8) | 1512 (53.9) |  |
| Only child (%) | |  |  | 0.688 |
|  | No | 87 (87.0) | 2385 (85.0) |  |
|  | Yes | 13 (13.0) | 420 (15.0) |  |
| Boarding (%) | |  |  | 0.588 |
|  | No | 34 (35.1) | 895 (31.9) |  |
|  | Yes | 63 (64.9) | 1910 (68.1) |  |
| Parents’ marital status (%) | | |  | 0.945 |
|  | Married | 80 (80.8) | 2289 (81.6) |  |
|  | Other | 19 (19.2) | 516 (18.4) |  |
| Perceived family income (%) | | |  | 0.067 |
|  | Upper | 22 (21.8) | 390 (13.9) |  |
|  | Middle | 65 (64.4) | 2055 (73.3) |  |
|  | Lower | 14 (13.9) | 360 (12.8) |  |
| Depression Symptoms  (Md [P_25_, P_75_]) | | 5 [0, 10] | 5 [2, 9] | 0.358 |
| Negative Life Events  (Md [P_25_, P_75_]) | | 20 [9, 43] | 18 [9, 33] | 0.125 |
| Self-Esteem  (Md [P_25_, P_75_]) | | 27 [24, 31] | 27 [24, 30] | 0.941 |
| Coping Tendency  (Md [P_25_, P_75_]) | | -0.36 [-0.95, 1.03] | -0.02 [-0.89, 0.87] | 0.619 |
